# Construction of recombinant fusion protein of influenza, a virus neuraminidase and heat shock protein 70 gene: expression in baculovirus and bioactivity

**Published:** 2015

**Authors:** M Moghaddam Pour, H Keivani, SH Masoudi, SH Monavari, M Najafi

**Affiliations:** * Virology Department, School of Medicine, Iran University of Medical Sciences, Tehran, Iran; ** Research and Development Viral Vaccine Department, Razi Vaccine & Serum Research Institute, Karaj, Iran; *** Poultry Viral Vaccines Research Department, Razi Vaccine & Serum Research Institute, Karaj, Iran; **** Cellular and Molecular Research Center, Biochemistry Department, Iran University of Medical Sciences, Tehran, Iran

**Keywords:** influenza, reverse genetics, cloning, neuraminidase, heat shock protein (HSP)

## Abstract

**Background:** Two structural antigens, hemagglutinin and neuraminidase, are a major component for the development of influenza vaccine candidates. Recombinant vaccines are produced by a simple method, although expected to induce an immune response to a specific antigen, remaining to be further improved for their high effectiveness. In general, heat shock protein 70 of Mycobacterium tuberculosis, as a potent adjuvant, is commonly used to improve antigen-presenting cell (APC) function and thereby elicit T lymphocytes.

**Objective:** The purpose of this research was to evaluate the efficacy of the NA antigen fused to the C-terminus of HSP70, as a vaccine candidate, in the induction of potent, protective immune answers specific to the vaccine antigen.

**Material and Method:** The NA gene was strengthened via a polymerase chain reaction and then cloned to a eukaryotic expressing vector pFastBac HTA. Subsequently, a recombinant NA protein fusing to HSP70 was expressed in Baculovirus. The purity of the expressed NA-HSP70 fusion protein was investigated on the SDS-PAGE electrophoresis. Western blot was carried out to investigate the expression of NA-HSP70. Additionally, an immunofluorescence assay was used qualitatively to assess the biological and antigenicity activity profiles of the protein of recombinant, NA-HSP70, on the infected Sf9 cell surface by using immunized rabbit antiserum.

**Result and conclusion:** Interestingly, the findings in the present studies suggested that HSP proteins have the ability to both stimulate and increase potent humoral- and cell-mediated immune responses, and play an adjuvant role when combined with other proteins. Therefore, a recombinant protein fusing to HSP raised hope regarding the development of an HSP-based vaccine.

## Introduction

Influenza A virus has emerged as an important pathogen affecting a wide range of various animals [**1**]. Influenza viruses are related to Orthomyxoviridae and are classified into 3 kinds A, B, C. Influenza kind A virus is considered the primary subtype on high-virulence properties and the risk of potential epidemic development. The influenza genome includes eight negative sense RNA parts representing ten proteins. Neuraminidase (NA) and hemagglutinin (HA) glycoproteins, the main antigenic proteins of the viral core [**2**], seem to have the capability to elicit an immune reaction against the virus, leading to a protective immunity against flu disease. Currently, there are two licensed antiviral medications available to target the NA glycoprotein, which have been used successfully in treating patients with influenza. Also, several promising new therapies with NA profiles are under advancement. However, NA has been widely not seen as a vaccine goal versus the fact that the inclusion of NA in subunit vaccines can provide advanced safety. In recent years, the majority of studies focused on the NA glycoprotein because it was well demonstrated that the NA gene is more stable than the HA one [**3**], meaning that no post-translational cleavage occurs beyond the NA polypeptide processing [**4**-**6**]. It has been shown that the viral neuraminidase takes part in several main roles in the viral infection, such as the cleavage of sialic acid during the late stage of infection [**7**,**8**], as well as the replication and transmission of the influenza virus [**9**]. However, although NA is a kind for vaccine advancement versus the virus of influenza, Ze et al. showed that the use of NA-subunit vaccines alone failed to induce a complete protective immunity against the influenza virus [**10**]. To circumvent this problem, we used an efficient immune stimulator, Heat-shock proteins (HSPs) to elicit the protective immune responses against influenza. More recently, it was well documented that HSP70 derived from Mycobacterium tuberculosis, which has the ability to stimulate and increase the potent humoral and cell-mediated immune reactions that further display chaperone activities and adjuvant functions when combined with other proteins [**11**-**13**]. Also, it should be noted that the c-terminal domain of Mycobacterium tuberculosis HSP70 exhibits potent adjuvant properties, conferring a better stage of safety in front of the influenza virus. Given the above, the aim of current research was to survey the expression pattern of the NA gene fusing to HSP70, as a chimeric fragment, into an expression vector system, to design future recombinant viral vaccines [**14**]. The adaptation of a baculovirus expression system to develop the ex-vivo, vivo, and in-vitro, in the production of functional eukaryotic proteins in mammalian cells, makes it a promising alternative to vectors based on human viruses [**15**]. More importantly, the NA-HSP70 fusion gene, expressing a fusion protein, provides a scope for further studies, to pave the way for the future developments of recombinant protein vaccines [**16**].

## Materials and methods

**Construction and Cloning of a Shuttle vector**

**Fig. 1:**
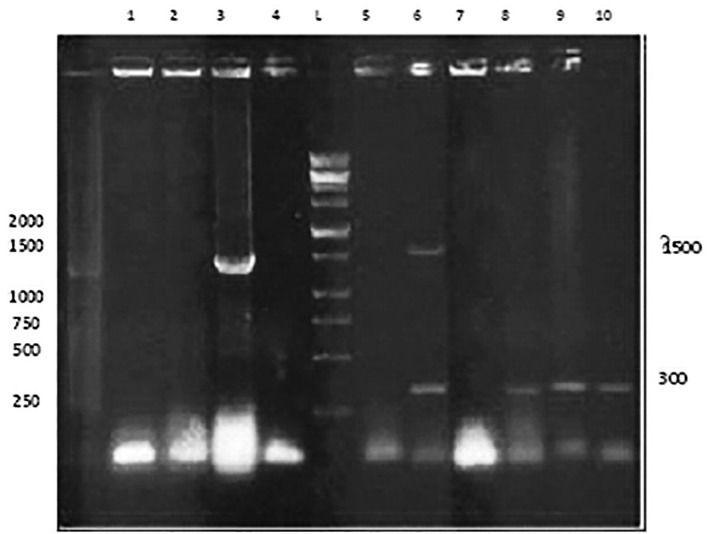
PCR analysis of the pFastBacNA integrant. The integration of NA into the pFastBac-HTA plasmid was checked by colony PCR screening by using specific pFastBac-HTA primers. The pFastBacNA integrant contained two different bands of approximately 1500 and 300 bp, indicating that the NA gene was integrated into pFastBac-HTA. Lane L: a 100-bp DNA ladder, lane 6: the pFastBacNA amplicon, lanes 8 to 10: an empty pFastBac-HTA vector, lane 3: the NA gene as a positive control, and lanes 1,2,4,5 and 7: inserted Top10 as a negative control

**Fig. 1** represents the pFastBac HTA vector, containing an N-terminal gp67 sign peptide, a thrombin fracture section, a His6 tag, and an N-terminal tetramerization zone [**9**]. The strains of influenza A virus (A/Brisbane/59/2007(H1N1)) were gained from WHO and propagated in embryonated chicken eggs. The ectodomain, including the stalk region (positions 37 to 469) of a nearly full-length NA protein is available in the NCBI protein database under the following accession number: gi|292486392|gb|CY058489.1|. PCR primers amplification was designed with overhanging Kpnl & HindIII restriction enzyme cutting sites (1437 bp). M. Tebianian kindly provided the HSP70 gene (GenBank: ACE79189.1) deriving from Mycobacterium tuberculosis 37Rv cloned into pQE30 vector. The primers designed for the HSP70 gene and the two restriction enzyme cleavage sites (Bamh1 & HindIII, 823 bp) were artificially introduced into two pairs of primers. Also, a restriction site for Kpnl was located on the nucleotide position 535. The N-terminal 6×His tag and the AcTEV protease recognition site were inserted downstream of the polyhedrin promoter first; NA was cloned in the correct reading frame with the initiation of ATG codon at nucleotides 4050 to 4052. The first open reading frame (ORF) of the virus encoded the NA glycoprotein (1437 bp). HindIII and Kpnl restriction sites introduced in each primer facilitated the double restriction enzyme digestion of the vector pFastBac HTA throughout the same limitation parts needed to build a pFastBacNA plasmid [**15**]. Afterwards, the HSP70 gene was cloned into pFastBacNA. HSP70 and purified pFastBacNA were recovered by Bamh1 and Kpnl second restriction enzyme digestion to produce the pFsBbacNAHsp70 (1030 bp) donor plasmid. 

**Construction of a Recombinant Bacmid**

**Fig. 2:**
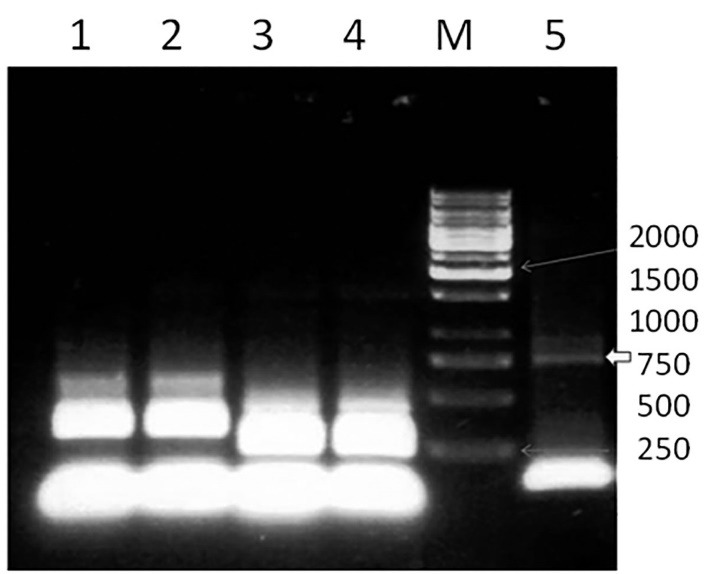
PCR analysis of the HSP70 gene. HSP70 was increased by using specific HSP70 primers. PCR amplification of HSP70 gene yielded a nearly 750-bp fragment. Lane M: a 100-bp DNA marker, lane 5: the HSP70 gene (sample 5), and lanes 1-4: negative controls

The RNA Kit (QIAGEN, Chatsworth, CA) and One-Step RT-PCR kit (QIAGEN) were utilized to extract vRNA and to perform the vice versa transcription-PCR (RT-PCR), respectively, according to the standard protocol. Universal textbooks were used to amplify the region of interest [**17**]. The target PCR products were excised from agarose gels and purified by using the QIA quick gel extraction kit (QIAGEN) [**18**]. The primers used to boost the NA region were the ones mentioned below: forward 5’- TTATATTTAGGTACCAAA TCAAA AGATAATAACC (Forward primer including a KpnI site), and vice versa 5’-TATTAATTTTAAGCTTCAAC GAAC T ACTT GTCAATGGTG (Reverse primer containing a HindIII section). The PCR products of NA were analyzed by 1% agarose gel electrophoresis and purified by using a QIA fast Gel Extraction kit (QIAGEN, Valencia, CA, and the USA). Subsequently, the NA fragment and pFastBac-HTA vector were digested with HindIII and KpnI to construct the pFastBacNA plasmid by T4 DNA Ligase. The resultant pFastBacNA plasmid transformed into bacterial cells (E. coli Top10 strain) by using the heat shock method [**11**,**19**]. Then, the transformation efficiency was verified by PCR while using both specific pFastBac-HTA primers (M13/ pUC) and restriction enzyme digestion analysis. As previously stated, pFastBac-HTA was amplified by using two specific primers (forward 5’-TATTCCGGATTATTCATACCGTC, and reverse 5’- GTATGGCTGATTATGATCCTC). The findings were then investigated through a 1% agarose gel electrophoresis [**15**]. The HSP70 gene was inserted into the pFastBacNA plasmid. The primers used to amplify the HSP70 gene were the following: forward 5’-TTATATTTAGGATCCAGAGGTGAAAGACGTTATGCTGC (forward primer including a BamhI restriction section), and vice versa 5’- TAATTAATAACGGA CCGAAG CTTGGCC TCCCGGCCGTCGT (Reverse primer including a HindIII restriction section). The PCR fragment of HSP70 was extracted by 1% agarose gel electrophoresis and purified by using a QIA fast Gel Extraction pack (QIAGEN, Valencia, CA, and the USA). Subsequently, the HSP70 gene was cloned into the pFastBacNA vector, followed by the transformation in E. coli Top10 and DH10 competent cells. The recipient cells were cultured in SOC solid media containing Kanamycin (50µg/ ml), Tetracycline (10), Gentamicin (7), X-gal (100) and IPTG (40) µg/ ml, and incubated at 37 ˚C for 48 hours [**20**,**21**]. The recombinant bacmid DNA was extracted from the culture pellets based on guidance provided through the constructor [**15**]. The transformation efficiency was verified by the PCR analysis by using a combination of M13/ pUC primers, as mentioned above, and double digestion with HindIII and BamhI [**15**,**20**,**22**-**24**] (**Fig. 2**).

**Transfection of Sf9 cells**

Sf9 cell lines, Spodoptera Frugiperda, were grown in Schneider’s cell culture medium. To construct the baculovirus recombinant expressing the NA-HSP70 fusion protein, the purified bacmid DNA was transmitted to Sf9 cells by using Lipofectamine, according to Invitrogen’s instructions [**15**,**25**]. Serum- and antibiotic-free medium (Un-supplemented necessary Schneider’s cell culture media) was used in this case [**15**,**20**,**22**]. A recombinant baculovirus was constructed when transfected cells started to grow. Cell culture was monitored daily for cytopathic effects (CPE). After 96 hours of cell culture, the medium was harvested and centrifuged at 1500 RPM, for 5 minutes. The recombinant performance was passed three times simultaneously.

**Expression of the recombinant protein**

Sf9 cell behaved with the recombinant performance at a multiplicity of infection (MOI) equal to 10, then it was incubated at 27°C ± 0.5 for 72 hours and monitored daily. Supernatants from the infected cultures harvested, and the fresh medium were added at different times. The medium removed and the attached cells expressing the protein of interest resuspended in the buffer containing 0.1 M Tris (6.8 PH) and 2% SDS. After the removal of the cell debris by centrifugation at 9500 rpm, up to a quarter hour, the cleared supernatants were stored at -20°C [**15**,**22**].

**Immunofluorescent assay**

Following baculovirus infection of Sf9 cells, foci of CPE were detected in the infected cells, fixed with cold methanol at ambient condition for 600 seconds, next being rehydrated at 37oC up to 1 hour. The infected cells were incubated at 37oC, up to one hour, with the rabbit polyclonal antibody against NA, cleaned by PBS three times, and incubated via the FITC-conjugated anti-rabbit secondary antibody. Bound antibodies were detected by the fluorescein-labeled antibody. Finally, Fluorescein-labeled cells were observed and photographed under a fluorescence microscopy [**26**-**29**].

**SDS- PAGE & Western blot evaluation**

The protein was denatured with sodium dodecyl sulfate ion Detergent (SDS). The samples were loaded on 12.5% SDS-PAGE gel to separate proteins according to their molecular weight [**30**]. The membrane blocked with 2% BSA in TBS buffer and after washing with TBS-tween-20, the membrane was reacted with rabbit polyclonal antibody specific to NA for two hours in ambient condition. Regarding the next cleaning, the membrane was reacted with anti-rabbit antibody HRP-conjugate for two hours at ambient condition and next cleaning the particular bands appeared with DAB substrate [**31**].

## Results

**Verification of the Recombinant Bacmid**

**Fig. 3:**
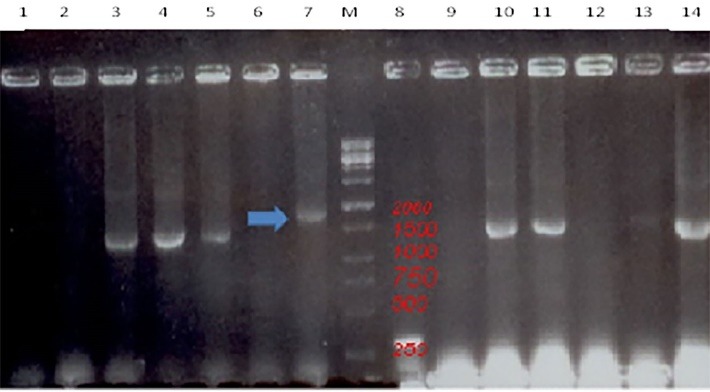
PCR analysis of the pFastBacNA-HSP70 plasmid in Top 10 cells was carried out by using specific pFastBac HTA primers (the Screening test). The pFastBacNA-HSP70 plasmid exhibited a nearly 1800-bp fragment. Lane M: a 100 bp DNA marker, lane 7: the amplicon of pFastBacNAHSP70 (active colony). Lane 8: an empty pFastBac-HTA vector indicating a 303-bp fragment, lanes 3,4,5,10,11,13 and 14: Top 10 cells colonies containing only the pFastBacNA plasmid, but no accepted HSP70 gene to show the 1500-bp fragment, lanes 1,2,6,9 and 12: empty Top10 cells as a negative control without the fragment

Following the amplification of the NA gene, the specific fragment of approximately 1400 bp carrying the NA gene was detected by 1% agarose gel electrophoresis, as shown in **Fig. 3**. After the transformation of Top10 strain cells, the presence of the pFastBacNA plasmid was verified in culture-positive samples via PCR by using M13/ pUC predefined primers. **Fig. 1** depicts vectors with and without the NA-HSP70 gene and represented a 303bp fragment in the PCR analysis.

Restriction endonuclease analysis was carried out by using KpnI and HindIII restriction enzymes to confirm the presence of the correct insert. The digested product produced 1500-bp fragments. Following the transformation of Top10 and DH10 cells with the pFstBacNA-HSP70 plasmid, the bacmid was constructed by transposons flanked by the insert region, in the area containing the LacZα gene for the annealing of M13/ pUC particular DH10 primers to the template. After the blue-white selection, positive white colonies were picked up and screened according to the manufacturer’s instructions [**15**]. As illustrated in **Fig. 2**, based on the position and length of the inserted NA-HSP70 fragment in the bacmid, an 1800-bp fragment was found to be amplified in PCR using M13/ pUC specific primers. 

Transfection was verified by PCR analysis by using M13/ pUC primers, and double digestion with HindIII and BamhI, as depicted in **Fig. 3**, respectively.

**Transfection of insect cell lines**

**Fig. 4:**
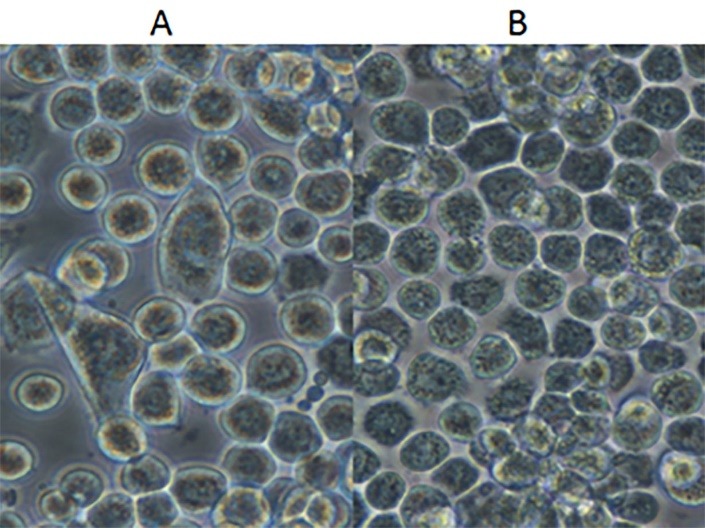
A) Sf9 cells without DNA transfection as a negative control. B) Transfected cells with recombinant bacmid DNA presenting typical CPE resulted from baculovirus propagation

Following transfection of the pFastBacNA-HSP70 bacmid into Sf9 cells, viruses propagated in the culture were found to develop cytopathic effects (CPE) in Sf9 cells, i.e. enlarged cells, division stop (**Fig. 4**).

**Immunofluorescent assay**

**Fig. 5:**
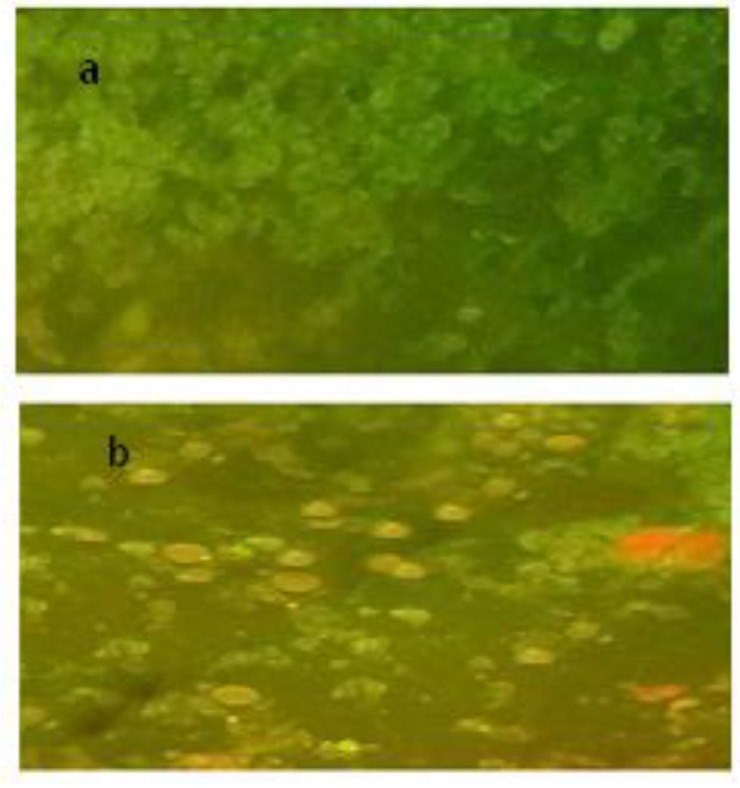
The immunofluorescent assay of Sf9 cell surfaces expression of the recombinant NAHSP70 protein. Immunofluorescent reactivity of the anti-NA polyclonal antibody observed in a dark field, A: non-transfected Sf9 cells as a control. B: Sf9 cells infected via baculovirus including pFastBacNA-HSP70

Cell area extension of the NA-HSP70 fusion protein was observed in Sf9 cells infected via Baculovirus (pFastBacNAHSP70) at an infection multiplicity of 10 by using an indirect immunofluorescence assay. The NA-HSP70 fusion protein, expressed on the Sf9 cells, was detected by the particular anti-NA polyclonal antibody (**Fig. 5 A**). As shown in **Fig. 5 B**, no reactivity of anti-NA polyclonal antibodies was found in non-transfected Sf9 cells.

**Confirmation of the recombinant protein**

**Fig. 6:**
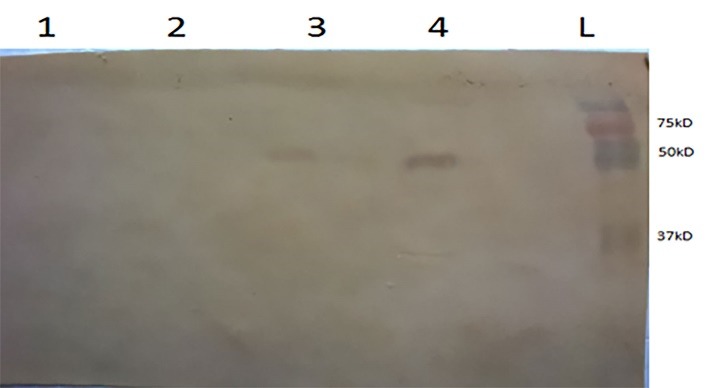
Western blot assay of the NAHSP70 protein expressed in Sf9 cells infected by baculovirus containing pFastbacNAHSP70. Lane 1: Non-transfected Sf9 cells, Lane 2 baculovirus containing pFastBacNAHSP70 (passage 2), Lane 3: culture supernatant of cells infected by pFastBacNAHSP70, Lane 4: infected Sf9 cells, and Lane L: a protein marker (Bio-Rad Ladder: Kaleidoscope Prestained Standards (161-0324))

The Sf9 cells infected with Baculovirus carrying the pFastBacNAHSP70 bacmid were harvested after 96 hours. The cells were lysed, and electrophoresis separated the total proteins at 12.5% SDS-PAGE and after a transfer to the membrane, by using a specific antibody the protein being characterized. The recombinant protein band with a molecular weight of approximately 49 kD showed NA-HSP70. As shown in **Fig. 6**, the Western blot displayed the specificity of the anti-NA polyclonal antibody directed against the band of interest. As a negative control, no band was detected in the non-transfected Sf9 cells.

## Discussion

Vaccination strategy as a possible approach saved millions of peoples from the infection disease. In this strategy for vaccine design, ideal molecules should be considered as vaccine candidates targeting and abrogating the disease [**9**,**16**,**32**]. Due to having an NA catalytic site, in this study, it was tried to have the full length of NA (an amplicon of approximately 1400 bp) to increase the efficacy of the immune response to the neutralization of the function of this protein in practice. Furthermore, it has been known that the catalytic site of NAs from some avian influenza viruses, unlike human influenza viruses, have a separate hem-adsorption site [**9**,**11**,**33**-**36**]. It is vital to consider that the NA protein stability has a main effect on the induction of immune responses. Simply stated, immune responses against both HA and NA glycoproteins usually protect against influenza infection. Nevertheless, some recent studies demonstrated that the NA gene is more stable as compared to the HA gene. On the other hand, it is well-known that the cell-mediated immune parameters, including IgG2a, Th1-kind (IFN-γ) and Th2-kind (IL-4) cytokines, play a critical role in virus clearance, but conventional inactivated influenza vaccines commonly lack the ability to induce cytotoxic T-lymphocyte (CTL) responses [**1**]. Interestingly, LAIV is the only vaccine that contains influenza-specific CD4 (+), CD8 (+), and γδ T cells for highly conserved influenza peptides [**37**]. In a study carried out by Dabaghian et al. it was reported that the heat shock protein 70 (HSP70) works as a natural adjuvant, when fused to 4 tandem periods of influenza A virus M2e, being able to skew T lymphocyte responses toward Th1 pattern in Balb/ C mice [**38**]. It was stated that HSP 70 could serve as a molecular target for the treatment of hepatocellular carcinoma (HCC) when overexpressed in hepatitis C virus (HCV)-related HCC [**39**,**40**]. In many studies, it has been shown that HSP-70 could result in the maturing of the dendritic cells as a key player in the induction of Th1 pattern. The activation of dendritic cells is a critical step of immune response for antigen presentation to T lymphocytes and its activation [**40**]. So, according to other experiences on vaccines formulated with HSP-70, here HSP-70 was used to improve the cellular immune response in parallel to humoral response for influenza vaccine candidate based on HA and NA proteins.

More recently, a variety of eukaryotic defining procedures, like yeast or insect cells, has been introduced as appropriate models to protein expression [**16**]. Because they are not replicated in mammalian cells, Baculoviruses are considered a convenient and safe host-vector system both in the in vitro and in vivo conditions. In recent decades, a particular consideration has been paid to the improvement of Baculovirus vectors, because of their high-capacity and biosafety profiles. Also, a Baculovirus expression system exhibited the ability to produce functional eukaryotic proteins efficiently in an extended variation of mammalian cells in vitro, ex vivo, and in vivo, providing a potential alternative to vectors based on human viruses [**15**]. In the present study, the seasonal strain of influenza virus (A/Brisbane/59/2007(H1N1)) was propagated in the embryonated Specific Pathogen-Free (SPF) eggs, followed by the amplification of the full-length gene by the specific primers. Next, the boosted was provided cloned into the pFastBac HTA vector. To construct a recombinant vector expressing a true protein of folded via a proper conformation and high immunogenicity, the NA gene fused to the N-terminus of Mycobacterium tuberculosis HSP70 (359-610), as an effective adjuvant. The newly synthesized vector was successfully cloned into the Baculovirus expression procedure. When cloned into the pFastBac HTA vector, the fusion gene was transfected and expressed in the Sf9 cells. Therefore, we constructed a plasmid vector carrying the NA-HSP70 genes, which is likely to be a potential vaccine candidate against influenza [**40**]. The expressed NA-HSP70 fusion protein was analyzed on SDS-PAGE, considered via Western blotting investigating the protein expression pattern by using the commercial anti-NA polyclonal antibody. The immunofluorescence assay was carried out qualitatively to detect the antigenicity and biological activity profiles of the recombinant protein (NA-HSP70). However, in the present study, NA-HSP70, the fusion protein was expressed, and bioactivity of this molecule was determined, but the immunogenicity and potency of this vaccine candidate remained to be clarified in the future on an animal model. 

**Acknowledgments**

We would like to thank Dr. Morteza Taghizadeh, Dr. Ali Reza Tavangar, Dr. Ali Ameghi, Miss. Mahdieh Jafari, Dr. Hadi Fazel, Miss. Zahra Bayat for technical assistance. Also, we would like to thank Dr. Mehdi Mahdavi for technical assistance and manuscript revision. This research was supported via a grant from Razi Vaccine and Serum Research Institiute, Karaj, Iran.
